# Association of the Neighborhood Retail Food Environment with Sodium and Potassium Intake Among US Adults

**DOI:** 10.5888/pcd11.130340

**Published:** 2014-05-01

**Authors:** Sophia Greer, Linda Schieb, Greg Schwartz, Stephen Onufrak, Sohyun Park

**Affiliations:** Author Affiliatons: Linda Schieb, Stephen Onufrak, Sohyun Park, Centers for Disease Control and Prevention, Atlanta, Georgia; Greg Schwartz, Veteran’s Health Administration, Seattle, Washington.

## Abstract

**Introduction:**

High sodium intake and low potassium intake, which can contribute to hypertension and risk of cardiovascular disease, may be related to the availability of healthful food in neighborhood stores. Despite evidence linking food environment with diet quality, this relationship has not been evaluated in the United States. The modified retail food environment index (mRFEI) provides a composite measure of the retail food environment and represents the percentage of healthful-food vendors within a 0.5 mile buffer of a census tract.

**Methods:**

We analyzed data from 8,779 participants in the National Health and Nutrition Examination Survey, 2005–2008. By using linear regression, we assessed the relationship between mRFEI and sodium intake, potassium intake, and the sodium–potassium ratio. Models were stratified by region (South and non-South) and included participant and neighborhood characteristics.

**Results:**

In the non-South region, higher mRFEI scores (indicating a more healthful food environment) were not associated with sodium intake, were positively associated with potassium intake (*P* [trend] = .005), and were negatively associated with the sodium–potassium ratio (*P* [trend] = .02); these associations diminished when neighborhood characteristics were included, but remained close to statistical significance for potassium intake (*P* [trend] = .05) and sodium–potassium ratio (P [trend] = .07). In the South, mRFEI scores were not associated with sodium intake, were negatively associated with potassium intake (*P* [trend] = < .001), and were positively associated with sodium–potassium ratio (*P* [trend] = .01). These associations also diminished after controlling for neighborhood characteristics for both potassium intake (*P* [trend] = .03) and sodium–potassium ratio (*P* [trend] = .40).

**Conclusion:**

We found no association between mRFEI and sodium intake. The association between mRFEI and potassium intake and the sodium–potassium ratio varied by region. National strategies to reduce sodium in the food supply may be most effective to reduce sodium intake. Strategies aimed at the local level should consider regional context and neighborhood characteristics.

## Introduction

High sodium intake and low potassium intake can contribute to increased risk of hypertension and cardiovascular disease (1–3). For adults, current guidelines recommend sodium intake of less than 2,300 mg per day with further reductions in specific subgroups and potassium intake of at least 4,700 mg per day (4). On average, US adults consume about 3,600 mg per day of sodium and 2,800 mg per day of potassium (5,6). The high levels of sodium intake and low levels of potassium intake have largely been attributed to consumption of processed foods such as bread and rolls, cold cuts and cured meats, and pizza (7). Studies have also suggested that the ratio of sodium intake to potassium intake may contribute to cardiovascular disease risk (8).

Many studies in the United States have examined various aspects of the food environment in relation to health outcomes. Studies have shown that the availability of healthful foods varies by retail food store type. Large supermarkets have a larger supply of produce and more healthful food choices whereas convenience stores and fast food restaurants tend to have fewer healthful food choices (9–12). Many studies have found that the presence or density of supermarkets and other healthful-food stores is associated with meeting dietary guidelines and eating more fruits and vegetables (13–16). However, none of the US studies have examined the relation between the retail food environment and sodium or potassium intake. A Japan study examined the association between retail food environment and urinary sodium and potassium excretion (17) and found an inverse correlation between the number of confectionary stores and bakeries (considered less healthful) and urinary excretion of potassium and the sodium–potassium ratio (17).

Given the current emphasis on community-level interventions for reducing sodium consumption (18), examination of the association between neighborhood retail food environment and sodium and potassium intake will inform public health practitioners on how local context may influence patterns of sodium and potassium intake. To examine this question, it is necessary to link measures of the retail food environment with an individual’s dietary intake data. This linkage is possible with the National Health and Nutrition Examination Survey (NHANES). Our study assessed the relationship between the retail food environment and sodium intake, potassium intake, and the sodium–potassium ratio by using a nationally representative sample of US adults aged 20 years or older.

## Methods

### Data sources

We conducted a cross-sectional analysis of 8,779 participants aged 20 years or older who completed at least a single day of 24-hour dietary recall in NHANES, 2005–2008. NHANES is a multistage probability survey that collects data on health and nutrition indicators from adults and children in the United States. A subsample of NHANES participants completed at least 1 dietary recall via in-person interview of all foods consumed within a 24-hour period.

We obtained 2005–2009 US Census tract-level American Community Survey estimates for the following variables: percentage living in poverty, population density, and percentage white population. We also used standard US Census definitions to classify metropolitan status (urbanized area, urban cluster) and region (Northeast, South, Midwest, West). Census tract was used as a proxy for neighborhood and will be referred to as “neighborhood” in this article.

### Outcome variables

Trained interviewers used the US Department of Agriculture (USDA) automated multiple-pass method to collect information on all foods and beverages consumed within a 24-hour period. NHANES provides estimates of total daily sodium and potassium consumption based on sodium and potassium content of individual foods reported and salt used in cooking, added at the table, and contained in supplements and medicines (19).

To account for day-to-day variation in nutrient intakes, we predicted the participant’s usual intake of sodium and potassium by using a method developed by the National Cancer Institute (8,20). The sodium–potassium ratio was then calculated as the ratio between these predicted values.

### Exposure variables

We measured the retail food environment by using the modified Retail Food Environment Index (mRFEI) (21). Of the total number of food retailers in a census tract and the 0.5 mile buffer surrounding the census tract, the mRFEI represents the percentage of retailers that are more likely to sell healthful food. The equation for the mRFEI is shown below:

mRFEI = × 100

The mRFEI bases store classifications on the North American Industry Classification System codes. The mRFEI defines healthful-food retailers as supermarkets and larger grocery stores, supercenters, and produce stores (which include stands and markets that sell fruits and vegetables). All data on supermarkets, supercenters, and produce stores were obtained from the InfoUSA business database, 2009.

The mRFEI defines less healthful- food retailers as convenience stores, smaller grocery stores with fewer than 3 employees, and fast food restaurants. Convenience store data were obtained from the Homeland Security Information program database, 2008 (http://www.dhs.gov/infrastructure-information-partnerships); small grocery store data were obtained from the InfoUSA business database, 2009; and fast food restaurant data were obtained from the NavTeq database, 2009.

We classified mRFEI scores into 5 groups. To conduct comparisons with areas containing no healthful- food retailers, we considered mRFEI = 0 as a separate category. The remaining categories were 0 < mRFEI ≤ 7, 7 < mRFEI ≤ 10, 10 < mRFEI ≤ 18, and mRFEI > 18. These groups represent the proportion of healthful-food retailers within a half-mile radius of a census tract (ie, mRFEI > 18 represents census tracts with more than 18% of retailers classified as healthful-food retailers).

### Participant characteristics

Individual characteristics included age, race/ethnicity, sex, education, income–poverty ratio, and total energy intake (energy intake was collected from 24-hour-dietary recall by using the same automated multiple-pass method referenced for sodium and potassium). We classified age into 3 groups: 20 to 34 years, 35 to 64 years, and 65 years or older. We classified race/ethnicity into 4 groups: non-Hispanic blacks, non-Hispanic whites, Mexican American, and other. We classified education as less than high school completion, high school graduate, and some college or more. We assessed household income by using the income–poverty ratio, which represents the ratio of family income to the federal poverty level. We used a cut point of 1.3 for the ratio, which is consistent with the income requirement for the Supplemental Nutrition Assistance Program. We modeled total energy intake in kilocalories as a continuous variable.

### Census tract characteristics

The percentage of the population living in poverty, percentage white population, population density, and metropolitan status were census tract-level covariates in the model. We categorized percentage living in poverty (≤5%, 6%–19%, and ≥20%) and percentage white population (≤25%, 26%–86%, and >87%) by tertiles. We categorized population density (population per square mile) by quartiles: 416 or less, 417 to 2,767, 2,768 to 6,705 and greater than 6,705. We classified metropolitan status according to census urban definitions: urbanized area, urban cluster, or nonmetro status (if the tract was not in urbanized area or urban cluster). We also classified each tract by census region: South, Midwest, Northeast, and West.

### Statistical analysis

We calculated distributions of the study population by age, race/ethnicity, sex, income–poverty ratio, neighborhood (or tract) percentage white, percentage living in poverty, and population density, and we accounted for survey sampling weights. We also assessed mean differences in sodium intake, potassium intake, and sodium-potassium ratio by using *t* test and one-way analysis of variance. *P* values less than .05 were considered statistically significant. We used linear regression to examine the association between mRFEI and usual intake of sodium and potassium and the sodium–potassium ratio, which accounted for the complex sampling design of the NHANES survey. Our linear model results were compared with a linear mixed model that included a random effect for census tract. Results yielded the same conclusions, so we report the results of the linear model (without the random effect). We included the person’s age, race/ethnicity, sex, education status, income–poverty ratio, and total energy intake as covariates in the first model. The second model added neighborhood-level percentage white, percentage living in poverty, and population density. All models accounted for the complex NHANES survey sampling design. Statistical interactions between mRFEI and all covariates were assessed and showed a significant interaction by census region in which the association between food environment and sodium and potassium intake varied by region; subsequent tests for interaction by region showed that the association varied between South and non-South (Northeast, Midwest and West) census regions. Therefore, we stratified models by South and non-South. We also tested for linear trend for each model. All analyses were conducted using SAS 9.2 (SAS Institute Inc, Cary, North Carolina) and SUDAAN v10.0 (RTI International, Research Triangle Park, North Carolina). Because of restricted use of the census-tract–level data and to protect confidentiality, all analyses were conducted at the National Center for Health Statistics Research Data Center.

## Results

The distributions of participant and neighborhood-level characteristics are shown in Table 1. Most participants were younger than 65, were white, had a high school diploma or more, lived in households with income above 130% of the federal poverty level, and lived in neighborhoods classified as an urbanized area or urban cluster.

**Table 1:** Distribution of Individual and Neighborhood Characteristics of US Dietary Recall Participants Aged 20 Years or Older, by US Census Region, NHANES 2005–2008

Characteristic	Total		South		Non-South	
	n	Weighted % (95% CI)	n	Weighted % (95% CI)	n	Weighted % (95% CI)
**Age, y**						
20–44	3,672	46.9 (44.4–49.4)	1,505	47.5 (43.1–52.0)	2,167	46.6 (43.6–49.6)
45–64	2,903	36.0 (34.5–37.6)	1,181	36.0 (33.1–38.9)	1,722	36.1 (34.3–37.8)
≥65	2,204	17.1 (15.4–18.9)	858	16.5 (14.1–19.4)	1,346	17.4 (15.2–19.7)
**Race/ethnicity**						
Non-Hispanic white	4,322	71.9 (67.1–76.3)	1,341	61.9 (54.3–69.0)	2,981	77.4 (71.3–82.4)
Non-Hispanic black	1,948	11.4 (8.8–14.6)	1,200	19.6 (14.3–26.2)	748	6.9 (4.6–10.2)
Mexican-American	1,552	7.8 (6.2–9.9)	656	9.4 (5.9–14.7)	896	7.0 (5.5–8.8)
Other	957	8.9 (7.1–11.1)	347	9.1 (6.4–12.8)	610	8.7 (6.5–11.7)
**Sex**						
Male	4,436	48.3 (47.3–49.4)	1,799	49.1 (47.1–51.0)	2,637	47.9 (46.8–49.1)
Female	4,343	51.7 (50.7–52.7)	1,745	50.9 (49.0- 52.9)	2,598	52.1 (51.0–53.2)
**Education**						
Less than high school diploma	2,526	18.3 (16.2–20.7)	1,140	21.2 (17.7–25.1)	1,386	16.8 (14.1–19.9)
High school diploma	2,143	25.5 (23.6–27.5)	812	24.8 (22.2–27.6)	1,331	25.9 (23.3–28.7)
Some college or more	4,110	56.2 (52.5–59.8)	1,592	54.0 (49.5–58.5)	2,518	57.3 (52.1–62.3)
**Income–poverty ratio**						
≤1.3	2,485	19.5 (17.1–22.2)	1,079	22.2 (17.3–28.0)	1,406	18.1 (15.7–20.8)
>1.3	6,294	80.5 (77.9–82.9)	2,465	77.8 (72.0–82.7)	3,829	81.9 (79.3–84.4)
**Neighborhood, % white**						
≤25	2,089	12.2 (8.9–16.5)	1,069	17.5 (10.4–27.9)	1,020	9.3 (6.4–13.4)
25–87	4,454	53.3 (43.1–63.2)	2,116	68.9 (56.8–78.9)	2,338	44.8 (31.6–58.7)
>87	2,236	34.5 (24.9–45.6)	359	13.6 (6.9–25.1)	1,877	45.9 (32.0–60.5)
**Neighborhood, % people living at or below federal poverty level**						
≤5	2,432	36.1 (29.0–44.0)	782	29.0 (19.3–41.1)	1,650	40.0 (30.8–50.0)
5–19	4,716	51.5 (44.8–58.1)	1,983	55.0 (43.6–65.8)	2,733	49.6 (41.4–57.8)
≥20	1,631	12.4 (9.2–16.5)	779	16.1 (9.2–26.7)	852	10.4 (7.9–13.7)
**Neighborhood population density (population per square mile)**						
≤416	2,501	35.3 (24.9–47.4)	1,021	34.8 (20.7–52.3)	1,480	35.5 (22.0–51.8)
417–2,767	2,209	26.4 (20.6–33.1)	1,171	33.2 (22.7–45.6)	1,038	22.7 (16.2–30.9)
2,767–6,705	2,082	21.5 (16.8–27.2)	982	24.0 (16.6–33.3)	1,100	20.2 (14.5–27.5)
>6,705	1,987	16.8 (12.5–22.1)	370	8.0 (4.6–13.6)	1,617	21.5 (14.8–30.2)
**Neighborhood metropolitan status, US Census**						
Nonmetro	696	8.7 (5.1–14.4)	357	10.3 (5.1–19.7)	339	7.8 (3.6–16.2)
Urbanized area	2,019	27.7 (20.0–37.0)	824	27.0 (17.5–39.2)	1,195	28.1 (17.9–41.2)
Urban cluster	6,064	63.6 (52.9–73.1)	2,363	62.7 (46.4–76.6)	3,701	64.1 (49.8–76.3)
**Modified Retail Food Environment Index (mRFEI)**						
Least healthful mRFEI = 0	1,351	15.9 (11.8–20.9)	478	12.0 (6.4–21.3)	873	18.0 (13.0–24.3)
0 <mRFEI ≤7	1,519	15.5 (12.1–19.7)	607	18.4 (13.1–25.1)	912	14.0 (9.6–20.0)
7 <mRFEI ≤10	1,496	15.4 (11.5–20.2)	585	15.4 (10.3–22.4)	911	15.4 (10.4–22.2)
10 <mRFEI ≤18	2,546	28.3 (23.8–33.2)	1,143	31.3 (22.9–41.1)	1,403	26.7 (21.7–32.4)
Most healthful mRFEI>18	1,867	25.0 (19.8–31.0)	731	23.0 (14.9–33.8)	1,136	26.0 (19.6–33.6)

Abbreviations: NHANES, National Health and Nutrition Examination Survey; CI, confidence interval.

Sodium and potassium intake varied by social and demographic characteristics. Sodium intake tended to be highest among the youngest age groups, whites, men, and people with some college education or more, people with an income–poverty ratio greater than 1.3, and people living in neighborhoods with a predominantly white population, in both South and non-South regions (Table 2). Potassium intake was lowest among the oldest and youngest age groups (compared with the 45–64-y age group), non-Hispanic blacks, women, those with less than high school education, those with an income–poverty ratio at or below 1.3, and those living in neighborhoods with a low-percentage white population and 20% or more of residents living at or below the federal poverty level. The mean sodium–potassium ratio was highest among the youngest age group, non-Hispanic blacks, men, and those living in neighborhoods with 20% or more of residents living at or below the federal poverty level in both South and non-South regions. However, in the non-South region, the mean sodium–potassium ratio was highest among those with a high school diploma compared with those with less than high school completion and those with some college and more, and mean sodium–potassium ratio was highest among those living in neighborhoods with the lowest percentage white population.

**Table 2:** Mean Sodium Intake, Potassium Intake, and Sodium–Potassium Ratio Among US Adults Aged 20 Years or Older in South and Non-South US Census Regions, by Participant and Neighborhood Characteristics, NHANES 2005–2008

Characteristic	Sodium Intake, Mean (95% CI)	Potassium Intake, Mean (95% CI)	Sodium–Potassium Ratio, Mean (95% CI)
**South Region**			
**Age, y**			
20–44	3,607 (3,494–3,720)	2,551 (2,463–2,639)	1.45 (1.41–1.48)
45–64	3,380 (3,286–3,475)	2,703 (2,616–2,790)	1.28 (1.25–1.30)
≥65	2,791 (2,691–2,891)	2,482 (2,401–2,562)	1.15 (1.13–1.16)
*P* value^a^	<.001	<.001	<.001
**Race/ethnicity**			
Non-Hispanic white	3,470 (3,376–3,565)	2,711 (2,629–2,793)	1.30 (1.28–1.33)
Non-Hispanic black	3,222 (3,140–3,304)	2,221 (2,171–2,271)	1.48 (1.46–1.49)
Mexican American	3,193 (3,069–3,316)	2,560 (2,466–2,655)	1.26 (1.24–1.29)
*P* value^a^	.004	<.001	<.001
**Sex**			
Male	3,946 (3,867–4,025)	2,937 (2,865–3,010)	1.37 (1.35–1.40)
Female	2,855 (2,812–2,899)	2,264 (2,216–2,311)	1.30 (1.27–1.32)
*P* value^a^	<.001	<.001	<.001
**Education**			
Less than high school diploma	3,078 (2,964–3,193)	2,364 (2,290–2,438)	1.33 (1.31–1.36)
High school diploma	3,343 (3,237–3,448)	2,520 (2,441–2,598)	1.35 (1.33–1.37)
Some college or more	3,535 (3,464–3,605)	2,718 (2,641–2,796)	1.33 (1.30–1.36)
*P* value^a^	<.001	<.001	.37
**Poverty–income ratio**			
≤1.3	3,099 (2,990–3,209)	2,344 (2,273–2,415)	1.35 (1.31–1.39)
>1.3	3,474 (3,416–3,531)	2,665 (2,603–2,728)	1.33 (1.30–1.36)
*P* value^a^	<.001	<.001	0.25
**Neighborhood, % white**			
≤25	3,170 (3,078–3,261)	2,362 (2,283–2,440)	1.37 (1.31–1.43)
25–87	3,437 (3,368–3,506)	2,643 (2,586–2,700)	1.33 (1.30–1.35)
>87	3,439 (3,227–3,650)	2,645 (2,387–2,903)	1.32 (1.27–1.38)
*P* value^a^	<.001	<.001	.24
**Neighborhood, % people living at or below federal poverty level**			
≤5	3,504 (3,414–3,594)	2,778 (2,690–2,866)	1.28 (1.25–1.31)
5–19.9	3,457 (3,271–3,443)	2,537 (2,472–2,601)	1.35 (1.32–1.38)
≥20	3,301 (3,155–3,448)	2,459 (2,370–2,549)	1.37 (1.32–1.43)
*P* value^a^	<.001	<.001	.001
**Neighborhood population density (population per square mile)**			
≤416	3,386 (3,275–3,496)	2,584 (2,503–2,665)	1.34 (1.31–1.36)
417–2,767	3,372 (3,287–3,458)	2,603 (2,535–2,671)	1.32 (1.29–1.35)
2,767–6,705	3,443 (3,312–3,575)	2,591 (2,465–2,718)	1.36 (1.32–1.39)
>6705	3,330 (3,203–3,457)	2,611 (2,486–2737)	1.30 (1.24–1.37)
*P* value^a^	.71	.97	.02
**Neighborhood US Census metropolitan status**			
Nonmetro	3,454 (3,222–3,685)	2,606 (2,465–2,747)	1.35 (1.32–1.39)
Urbanized area	3,288 (3,162–3,414)	2,519 (2,464–2,575)	1.33 (1.28–1.38)
Urban cluster	3,424 (3,330–3,518)	2,624 (2,534–2,714)	1.33 (1.30–1.36)
*P* value^a^	.19	.09	.62
**Non-South Region**			
**Age, y**			
20–44	3,671 (3,623–3,718)	2,669 (2,613–2,725)	1.40 (1.38–1.42)
45–64	3,461 (3,370–3,553)	2,856 (2,770–2,941)	1.23 (1.21–1.26)
≥65	2,889 (2,843–2,935)	2,655 (2,602–2,709)	1.11 (1.08–1.13)
*P* value^a^	<.001	<.001	<.001
**Race/ethnicity**			
Non-Hispanic white	3,513 (3,455–3,571)	2,802 (2,737–2,867)	1.28 (1.26–1.30)
Non-Hispanic black	3,159 (3,052–3,266)	2,249 (2,184–2,314)	1.44 (1.40–1.47)
Mexican American	3,194 (3,116–3,271)	2,624 (2,551–2,696)	1.23 (1.22–1.25)
*P* value^a^	<.001	<.001	<.001
**Sex**			
Male	4,050 (3,993–4,106)	3,107 (3,051–3,162)	1.33 (1.31–1.35)
Female	2,917 (2,869–2,965)	2,391 (2,334–2,448)	1.25 (1.23–1.28)
*P* value^a^	<.001	<.001	<.001
**Education**			
Less than high school diploma	3,117 (3,028–3,207)	2,494 (2,424–2,563)	1.27 (1.25–1.30)
High school diploma	3,451 (3,370–3,531)	2,670 (2,606–2,733)	1.32 (1.29–1.35)
Some college or more	3,564 (3,514–3,614)	2,833 (2,779–2,887)	1.28 (1.26–1.30)
*P* value^a^	<.001	<.001	.003
**Income-poverty ratio**			
≤1.3	3,108 (3,028–3,189)	2,472 (2,381–2,563)	1.29 (1.25–1.33)
>1.3	3,537 (3,490–3,584)	2,791 (2,739–2,844)	1.29 (1.27–1.31)
*P* value^a^	<.001	<.001	.84
**Neighborhood, % white**			
≤25	3,210 (3,083–3,336)	2,474 (2,388–2,559)	1.33 (1.31–1.36)
25–87	3,491 (3,426–3,556)	2,751 (2,689–2,813)	1.29 (1.26–1.32)
>87	3,480 (3,388–3,571)	2,769 (2,681–2,858)	1.28 (1.26–1.31)
*P* value^a^	.002	<.001	.02
**Neighborhood, % people living at or below federal poverty level**			
≤5	3,565 (3,504–3,626)	2,821 (2,769–2,872)	1.29 (1.27–1.30)
5–19.9	3,419 (3,347–3,491)	2,713 (2,616–2,811)	1.28 (1.25–1.32)
≥20	3,247 (3,187–3,306)	2,497 (2,436–2,557)	1.34 (1.30–1.37)
*P* value^a^	<.001	<.001	.02
**Neighborhood population density (population per square mile)**			
≤416	3,476 (3,377–3,574)	2,795 (2,683–2,907)	1.27 (1.23–1.31)
417–2,767	3,547 (3,467–3,628)	2,790 (2,726–2,855)	1.29 (1.26–1.32)
2,767–6,705	3,424 (3,322–3,525)	2,682 (2,594–2,770)	1.31 (1.28–1.34)
>6,705	3,374 (3,291–3,457)	2,620 (2,563–2,677)	1.31 (1.29–1.34)
*P* value^a^	.01	.004	.19
**Neighborhood US Census metropolitan status**			
Nonmetro	3,461 (3,346–3,576)	2,709 (2,577–2,841)	1.30 (1.26–1.34)
Urbanized area	3,464 (3,342–3,587)	2,806 (2,671–2,942)	1.26 (1.22–1.30)
Urban cluster	3,457 (3,400–3,514)	2,705 (2,650–2,760)	1.30 (1.29–1.32)
*P* value^a^	.99	.32	.05

a *P* values represent *t* test or one-way analysis of variance test results.

Linear regression models showed that the mRFEI was not associated with sodium intake for either South or non-South regions after controlling for individual participant and neighborhood characteristics (Table 3). We observed a positive linear association between mRFEI and potassium intake in non-South regions when controlling for individual participant characteristics, and this association diminished when neighborhood characteristics were added to the model (Table 3). In the South region, an increasing mRFEI was mildly associated with a decrease in potassium intake, and this association also diminished when neighborhood characteristics were added to the model.

**Table 3:** Linear Regression Analysis of the Modified Retail Food Environment Index (mRFEI) and Sodium Intake, Potassium Intake, and Sodium–Potassium Ratio, NHANES 2005–2008

mRFEI^a^	South Region				Non-South Region			
	Model 1^b^, β (SE)	*P* Value	Model 2^c^, β (SE)	*P* Value	Model 1^b^, β (SE)	*P* Value	Model 2^c^> β (SE)	*P* Value
**Sodium, mg**								
Least healthful mRFEI = 0	1 [Reference]		1 [Reference]		1 [Reference]		1 [Reference]	
0 < mRFEI ≤ 7	35.4 (49.2)	.48	50.2 (41.2)	.23	−35.0 (32.6)	.29	−54.6 (29.0)	.07
7 < mRFEI ≤ 10	−16.3 (65.6)	.81	−8.5 (52.1)	.87	15.8 (36.7)	.67	−1.8 (39.3)	.96
10 < mRFEI ≤ 18	−16.1 (51.5)	.76	0.68 (49.3)	.99	15.7 (33.8)	.65	1.7 (25.6)	.95
Most healthful mRFEI > 18	−42.7 (46.0)	.36	−44.8 (56.9)	.44	4.6 (31.5)	.89	−1.6 (37.3)	.97
Trend test^d^	.20		.29		.45		.55	
**Potassium, mg**								
Least healthful mRFEI = 0	1 [Reference]		1 [Reference]		1 [Reference]		1 [Reference]	
0 < mRFEI ≤ 7	39.9 (36.1)	.28	27.0 (37.0)	.47	5.4 (45.7)	.91	−21.3 (38.8)	.59
7 < mRFEI ≤ 10	54.1 (75.4)	.48	51.8 (81.1)	.53	94.0 (31.4)	.005	71.8 (26.3)	.01
10 < mRFEI ≤18	−8.3 (30.4)	.79	0.46 (38.9)	.99	62.9 (25.1)	.02	42.3 (20.3)	.05
Most healthful mRFEI > 18	−84.4 (32.2)	.01	−70.3 (39.9)	.09	49.5 (21.6)	.03	37.8 (22.8)	.11
Trend test^d^	<.001		.03		.005		.05	
**Sodium–potassium ratio**								
Least healthful mRFEI = 0	1 [Reference]		1 [Reference]		1 [Reference]		1 [Reference]	
0 < mRFEI ≤ 7	−0.02 (0.02)	.35	0 (0.02)	.89	−0.02 (0.02)	.24	−0.02 (0.02)	.32
7 < mRFEI ≤ 10	−0.03 (0.03)	.32	−0.03 (0.04)	.40	−0.04 (0.02)	.01	−0.04 (0.01)	.008
10 < mRFEI ≤ 18	0 (0.01)	.79	0 (0.02)	.99	−0.03 (0.01)	.009	−0.03 (0.01)	.02
Most healthful mRFEI > 18	0.02 (0.01)	.08	0.01 (0.02)	.58	−0.02 (0.01)	.02	−0.02 (0.01)	.07
Trend test^d^	.01		.40		.03		.07	

a The mRFEI provides a composite measure of the retail food environment and represents the percentage of healthful-food vendors within a 0.5 mile buffer of a census tract.

b Model 1 controls for age, race/ethnicity, sex, education, income–poverty ratio, and total energy intake.

c Model 2 controls for model 1 plus neighborhood percentage non-Hispanic white, neighborhood percentage at or below the federal poverty level, population density, and metropolitan status.

d Test for linear trend; *P* value is reported.

The mRFEI was most often negatively correlated with the sodium–potassium ratio in non-South regions; linear trend tests were significant for the model that controlled for individual characteristics but attenuated when neighborhood characteristics were controlled for. There was a linear association between increasing mRFEI and increasing sodium–potassium ratio in the South region, but this association did not hold once neighborhood characteristics were included in the model.

## Discussion

Our study is the first to examine the association between the local retail food environment and sodium and potassium intake in a large population of more than 8,000 US adults. Although this is an aggregate-level study, the results inform practitioners about neighborhood factors, including the local retail food environment, that are associated with changes in average sodium and potassium intake. Results suggest that food environment improvements are associated with mild increases in potassium intake in non-South regions and mild decreases in potassium intake in the South region. The sodium–potassium ratio decreased with improvements in the food environment in the non-South region and marginally increased with food environment improvements in the South region. There were no changes in sodium intake with improvements in the food environment in any region. Our results also show that other neighborhood characteristics (eg, percentage living in poverty, percentage white population) reduced the associations observed.

Our findings are mostly consistent with a previous study in Japan (17). Both studies suggest that food environment influences diet in reference to potassium intake. However, the magnitude of the associations in our study was small, so the results should be interpreted with caution.

The associations observed between food environment and both potassium intake and the sodium–potassium ratio diminished when neighborhood characteristics (percentage living in poverty, percentage non-Hispanic white population, population density, and metropolitan status) were included. This suggests that these factors contribute to the association between food environment and potassium intake and the sodium–potassium ratio. In general, participants living in high-poverty neighborhoods and neighborhoods with a low-percentage white population consumed less potassium than participants living in neighborhoods with a low percentage of poverty and a high percentage of whites (Table 2). Other studies have shown that neighborhoods that have a high percentage of black or poor residents have less favorable food environments (22). These factors should be considered when targeting local areas for food environment interventions.

Our findings that the association between food environment and potassium intake and sodium–potassium ratio differed between the South and non-South regions could reflect regional differences in diets. Previous studies suggest that types of foods consumed or food preparation methods may differ by region (23–25). A study by Farley et al. determined that the food microenvironment within stores devoted less space to fruits and vegetables in Louisiana than in Los Angeles (12). Although the association of mRFEI with diet has not been examined previously, other studies have found that related metrics are associated with fruit and vegetable consumption (16). Thus, regional differences in the food microenvironment could account for the observed variation in the association between mRFEI and potassium intake by region (12). In some circumstances, factors such as transportation (eg, vehicle access) and acculturation may outweigh the composition of the retail food environment, and these factors may vary by region (26,27). We did not measure characteristics such as transportation or cultural differences in the relationships between food environment and potassium or sodium intake, but these factors may be important in understanding the regional variations in the association.

Our results showed no significant relationship between food environment and sodium intake. Most adults in the United States consume high amounts of sodium because of its high content in processed foods (6,7). Because processed and high-sodium foods are offered in many supermarkets (and in other stores we considered in this study to offer healthful foods), the presence of a supermarket does not guarantee that people will eat foods with lower sodium content. It may be that because high-sodium foods are so ubiquitous (7) the presence or absence of food stores selling more healthful foods does not influence levels of sodium consumption. In addition, some foods such as those containing whole grains and dairy products, which are generally considered healthful, can have high sodium content (7).

This study has several strengths. It is the first to examine the association between the local retail food environment and sodium and potassium intake in the United States. We used a large sample of more than 8,000 NHANES participants, which allowed us to control for characteristics of the individual participant and the neighborhood and to stratify by region. We also used a composite measure to characterize the food environment, which allowed us to incorporate data on the density of both stores selling healthful food and those selling less healthful food instead of examining a single type of retail food store at a time. The use of composite measures of the food environment such as the mRFEI rather than counts of various retailers offers several advantages, such as data reduction and capturing the complexity of food environments (28). Finally, we calculated the usual intake of sodium and potassium to account for day-to-day variation in nutrient intakes.

Our study had several limitations. First, mRFEI relies on data from a commercial database that is subject to some error because of potential misclassification of retail stores, location data error, and lag in the indication of current operating status; we did not have a second data source to validate retail store locations. Because of confidentiality rules concerning the locations from which NHANES participants were sampled, we were also unable to verify the existence of food retailers or food offerings through on-the-ground observation. Second, although we did not use the mixed model to account for clustering within census tract, we did account for the complex survey sampling design of the NHANES survey, which accounts for clustering within counties. Third, we also assumed that participants were classified into neighborhoods that represent where they are most likely to purchase food; that supermarkets, large grocery stores, and produce stores have more healthful food choices than other types of food stores; and that people shop closest to their place of residence. Although this may be true in general, future studies are needed to examine other factors that may influence the likelihood that people will consume less sodium and more potassium (eg, transportation method, shelf placement, preferences) (26,27). Fourth, this is an aggregate-level study, and we were unable to measure the immediate food environment around each participant’s place of residence. However, food environment is commonly measured at the census-tract level, so this limitation may have had minimal effects on our results (12–15). Last, the automated multiple-pass method used to obtain dietary data tends to underestimate sodium intake, especially as body mass index (BMI) increases (29). If people with high BMIs are unequally distributed across neighborhoods, this could mask an association between food environment and sodium intake. However BMI was not a significant confounder or effect-modifier in our models.

Our study addresses an important question on the association of the local food environment with sodium and potassium intake. However, more work is needed to understand where and how people purchase foods and other neighborhood-level barriers and facilitators to consumption of healthful foods. Sodium intake was not associated with the food environment in this study. Interventions aimed at reducing sodium consumption may be most effective at levels other than the neighborhood retail environment, such as national public health interventions like the Institute of Medicine recommendation to reduce sodium levels in the food supply (11). Because improvements in the local food environment were associated with both increases and decreases in potassium intake, depending on census region, we also conclude that public health strategies that improve neighborhood-level access to healthful foods by adding supermarkets or increasing buying power for healthful food options should consider both regional context and neighborhood context (30).
